# Supplementary material for: Are carbon emissions trading and green financial instruments synergistic? -Comprehensive quantitative research based on content analysis
Source: PLoS One. 2024 Mar 7;19(3):e0298601. doi: 10.1371/journal.pone.0298601 (PMC10919601; doi:10.1371/journal.pone.0298601)
Supplement: S1 Data — (ZIP) [file pone.0298601.s001.zip › Data/地级市Z_F工作报告.zip!/░▓╗╒/╨√│╟╩╨/└┤╫╘╣1⁄2╓┌║┼í╛┬φ┐╦╩2╛▌═°í┐_2014.docx]

2014年政府工作报告

市长 韩 军

各位代表：

现在，我代表市人民政府，向大会报告政府工作，请予审议，并请市政协委员和列席会议的其他同志提出意见。

一、2013年工作回顾

刚刚过去的2013年，面对错综复杂的形势，全市上下在市委的坚强领导下，认真学习贯彻党的十八大、十八届三中全会和习近平总书记系列重要讲话精神，深入落实省委、省政府的决策部署，克服诸多自然灾害带来的不利影响，以“五年并进”、“五城同创”为抓手，立足“干”、突出“抢”、抓好“实”，稳中求进、有效作为，实现了经济稳健增长、社会稳定和谐、各项事业稳步发展。

初步预测，全年地区生产总值850亿元，同比增长11.5%左右。财政收入157.8亿元，增长14.6%。规模以上工业增加值340亿元，增长15%左右。服务业增加值280亿元，增长10%左右。固定资产投资970亿元，增长20%以上。社会消费品零售总额305亿元，增长14.5%左右。进出口总额16亿美元，增长20%以上。城镇居民人均可支配收入22730元，增长11%左右；农民人均纯收入突破万元，达10350元，增长14.5%左右。全面完成节能减排年度任务。

过去的一年，受宏观环境的影响，我市经济指标增幅有所回落，但总体保持高于全省平均水平、好于去年位次的态势，绝大部分指标实现了争先进位。一年来，我们主要做了以下工作：

（一）扎实推进“五个年”活动，经济实力进一步增强。 “招商引资年”深入开展。全市新签项目900个，其中与央企合作项目16个、知名民企合作项目82个。亿元以上项目到位省外资金590亿元，居全省第3位。实际利用外资5.3亿美元，增长20%。加强市本级招商力量，项目质量稳步提高。全市新引进5亿元以上项目46个、10亿元以上15个。新引进战略性新兴产业项目数占比达42%，同比提高13个百分点。新签项目单体平均规模1.5亿元，创历史新高。“项目推进年”有力有效。在建亿元以上项目445个，完成投资500亿元以上，均超过预定目标。列入省"861"项目184个，投资完成率、开工率、竣工率均居全省前列。谋划储备亿元以上项目514个。累计争取中央和省项目补助资金超8亿元、国外贷款2.4亿美元，其中全国唯一的宣城开发区1.5亿美元世行贷款示范项目启动实施。绩溪抽水蓄能电站、宣城电厂二期、南漪湖风电场一期等重大项目开工建设。“工业突破年”成效明显。新增规模以上工业企业232户，总数达1091户，分别居全省第3和第5位；实现销售产值1450亿元，产值超亿元企业突破300户。五大支柱产业和战略性新兴产业产值占规模工业比重达到62%和16%，分别提高1.8和2.1个百分点。钢铁、铜加工产值占比较“十一五”末下降15个百分点。工业项目建设取得突破，完成投资500亿元，增长24%。494个项目列入省工业投资导向计划。53个亿元以上项目投产。中鼎发动机和郎溪中讯通电子产业园、广德慈兴科技产业园等一批重大项目开工建设。淘汰关闭落后产能企业44家，提前完成省下达的“十二五”计划。“城市建设年”稳步推进。开展新一轮城市总体规划及彩金湖生态新区、环南漪湖区域总体规划编制，新编、续编和增编规划183项，在全省率先编制完成城市空间特色规划，实现城市近期建设用地范围内控规全覆盖。市本级续建和新建项目117个，完成投资152.6亿元，其中基础设施及公共项目投资66亿元。续建、新建道路36条，竣工通车9条。宣中新校区建成使用。宛陵湖西湖景区基本建成。期盼多年的图书馆、规划馆、体育馆全面开工建设，主体工程接近完工。合工大宣城校区二期、北门棚户区改造等工程加快推进。宣城海关、出入境检验检疫局综合楼和国家宣纸及文房用品质检中心、市国防训练基地、市示范性综合实践基地等项目开工建设。彩金湖生态新区建设揭开大幕。县城和中心镇建设加快，城乡面貌进一步改观，城镇化率达48%以上。“交通建设会战年”高速起步。完成建设投资逾72亿元，超过“十一五”交通投资总和。新增高速公路46公里、一级公路61公里，改善国省道180公里、县乡道200公里，公路路况水平由全省靠后进入前五位。宣宁高速、宣狸一级公路建成通车，宁绩、宁千高速建设加快，宣南铜高速全面复工，溧广高速、宣泾线等一级公路和郎溪、宁国客运枢纽站开工建设。农村公路危桥改造任务超额完成。芜申运河宣州段完工，郎溪定埠段进展顺利。合福铁路宣城段站前工程基本完成，26座隧道顺利贯通，三县车站站房建设全面启动。皖赣铁路扩能改造工程芜湖至宣城段开工建设。要素保障不断强化。加强政银企对接，分别与省进出口银行、国开行、徽商银行签订战略合作协议，银行业新增贷款123亿元，再创历史新高，新增贷存比位居全省第一。融资性担保机构增资扩股1.7亿元。市国投公司15亿元公司债获得批准。6家企业在省股权交易中心挂牌或托管。江南化工短期融资券成功发行。努力保障重点项目用地需求，批准用地面积居全省前列。新建成标准化厂房突破150万平方米。创建国土资源执法和节约集约模范市（县）工作取得成效。加强供需对接、校企对接，缓解企业用工困难。

（二）扎实推进“五城”同创，城乡魅力进一步增强。省级文明城市创建成效显著。以解决群众反映强烈的问题为着力点，组织实施“七整治一提升”专项工作，市区环境卫生状况明显改善，城市文明形象彰显。完成城市道路“白改黑”7条，改造老旧小区230个，清淤截污河道7条，治理背街后巷和主次干道186条，整治集贸市场和菜市场21个，整治小餐饮1158家，新增和改建垃圾中转站7个，机械化清扫率提高15个百分点。市区89台燃煤锅炉改造基本完成。大力整治二手车市场，皖东南旧机动车交易市场投入使用。强化社会停车场建设和管理，开放部分单位停车场。加强公民思想道德建设，入选中国好人榜7人、安徽好人榜13人，评选宣城好人106人。国家生态市和环保模范城市创建步伐加快。宁国和绩溪成为全国生态县，泾县入选“中国绿色发展十强县”，郎溪、广德、泾县、旌德创建国家生态县已报环保部，新增国家级生态乡镇8个，省级生态乡镇10个、生态村12个。实行了机动车环保标志管理和高污染排放机动车区域限行。国家园林城市和森林城市创建取得成效。市区新建城市公园3个，建设和提升6条绿带，新增绿化面积140万平方米，建成园林式单位、小区108家。创建省级森林城市1个、城镇10个、村庄26个。新造林18.8万亩，建设森林长廊示范段80.5公里。美好乡村建设深入推进。扎实开展10条示范带建设，顺利完成35个省级重点示范村建设任务，整治中心村、自然村223个。绩溪龙川村入选全国首批美丽宜居村庄。农村垃圾处理水平进一步提高，95个村开展垃圾分类减量试点，42个乡镇、177个村建立市场化运行机制。完成农村危房改造1.97万户。城乡水利、电力、通信建设继续加强。水阳江开卡工程主体完工。双桥联圩宋墩排涝站试运行。完成青弋江灌区溪口闸、郎溪中斗闸改造和双桥联圩堤防除险加固一期工程。关庙水库开工建设。66座小型水库除险加固工程主体完工，农村安全饮水工程年度任务全部完成。12个中小河流治理项目顺利实施。小农水重点县建设全面展开。电网建设投入7亿元，居全省第5位。建成投产220千伏郎溪梅丰变等5项输变电工程，历经十年的110千伏市区中心变电站建成投运。广德500千伏变电站取得核准。加强“智慧城市”基础网络建设，3G网络实现全覆盖。

（三）扎实推进转型创新，内生动力进一步增强。以文化旅游产业为重点的现代服务业加快发展。文化旅游产业“八大工程”规划编制完成。第六届中国（宣城）文房四宝文化旅游节成功举办，并荣获“美丽中国·最具品牌价值文化艺术节”称号。新创4A级景区2家、四星级宾馆3家。敬亭山风景区建设管理持续加强。扎实推进乡村旅游发展三年行动计划，新创省级特色景观旅游名镇名村12个、乡村旅游示范村15个、四星级以上农家乐19家。泾县被评为全省休闲农业与乡村旅游示范县。郎溪入选中国“十大休闲小城”。国内外游客接待量、旅游总收入分别增长18%和15%。宣纸文化产业园入选全省十大文化产业园区。8家企业进入全省民营文化企业百强。文房四宝产业园建成开园。金融体系继续完善，全面完成农商银行改制，新增金融机构和网点8家。商贸市场持续繁荣，“五位一体”便民服务体系和“新网工程”建设稳步推进。国购广场全面开业。淘宝网宣城馆、宁国馆成功上线。在全省率先开展“家具下乡”试点。现代农业发展水平持续提升。新增省级龙头企业24家、农民专业合作社示范社11家，总数继续保持全省领先，发展家庭农场612个，郎溪家庭农场发展方式成为全国五种模式之一。宣州、广德被评为全省创建农民专业合作社示范县。创建国家级农业产业化示范区1个，省级农业产业化示范区1个、现代农业示范区3个。创建国家级畜禽标准化养殖小区2个、省级6个，农业部水产健康养殖场2家。农产品加工产值增长20%。新建良种茶园1.6万亩。烟叶、中药材种植面积分别达到12.3万亩和10万亩。泾县成功创建全省首家“全国有机农业（茶叶）示范基地”。宣州荣获“全国粮食生产先进单位”称号。认真执行粮食最低收购价政策，托市收购粮食12万吨。新建市区保障性蔬菜基地1000亩。科技创新能力显著增强。首次荣获“全国科技进步先进市”，5个县市区进入“全国科技进步先进县”行列。12个项目列入国家星火计划，5个项目列入国家火炬计划。新增国家火炬计划重点高新技术企业1家，新认定高新技术企业31家、省级创新型试点企业6家，新增省级高新技术产品143项，实现高新技术产业增加值117亿元，增长17.6%。新增省级“两化”融合示范企业6家。新增省级博士后科研工作站2个、工程技术研究中心4家、企业技术中心6家。新组建企业院士工作站1家。新增发明专利授权量85件，增长30.8%。在全省率先出台《质量发展纲要》。主导、参与制修定国家、行业、省地方标准30项。3户企业获得省质量奖。新增省著名商标26件、专业商标品牌基地2个、安徽名牌产品15个。园区转型发展步伐加快。宁国开发区成功升格国家级。宣城、广德开发区成为全省产城一体化试点园区。宣州、郎溪、广德开发区扩区获批。宣州、宁国开发区获批省级新型工业化产业示范基地。广德开发区获批省级电子信息产业基地。港口生态工业园区跻身国家智慧城市试点。双桥物流园区获批省级服务业集聚区。民营经济进一步壮大。民营经济增加值占生产总值比重较上年提高1个百分点。新注册私营企业2843户、个体工商户15150户。9家企业进入全省民企百强。新增省级小微企业创业基地6家、“专精特新”企业11家、产业集群专业镇5个。改革试点稳步推进。宣城成为全国中小商贸流通企业服务体系建设试点城市。广德成为全省服务业综合改革试点县。在全省率先试行地票交易制度，率先开展森林保险试点。省级农民文化乐园建设试点工作取得初步成效。郎溪、广德列入国家知识产权强县工程试点县。宁国试行农村宅基地“有偿退出”。

（四）扎实推进社会建设和民生改善，发展合力进一步增强。民生支出占财政支出的81%；投入28.2亿元，完成省定33项民生工程。就业和社会保障工作得到加强。城镇新增就业5.98万人，城镇登记失业率3.25%。超额完成五项社会保险年度任务。将符合条件的被征地农民纳入城镇职工基本养老保险保障范围。开工建设各类保障性安居工程21319套，基本建成10675套，竣工7193套，建设进度位居全省前列。市本级开工建设安置房8251套，竣工3703套。商品住房价格基本稳定。市社会福利中心建成使用。新改扩建农村敬老院24所。马山生态陵园殡仪馆主体工程完工。文教卫体事业发展加快。公共文化场馆全部免费开放。村级文化广场建设列为国家公共文化服务体系示范项目。新增国家文物保护单位9处。泾县黄田古村落整体保护成为全国文物维修保护样板示范工程。绩溪仁里村、宁国胡乐村被列为第二批中国传统村落。全省最大的县级博物馆--绩溪博物馆落成开馆。210所义务教育学校完成标准化建设。学前教育三年行动计划目标基本实现。宣城职教联盟成立。县级公立医院改革扎实推进。疾病防控、卫生应急等公共卫生服务不断加强。宣城中心医院被批准为三级综合医院。成功举办市首届全民健身运动会。人口计生等工作取得新进步。出生人口政策符合率、出生人口性别比等指标继续保持全省领先。完成了“十二五”发展规划中期评估工作。妇女、儿童、老龄、残疾人和红十字、慈善福利事业迈出新步伐。统计、民族宗教、物价、外事侨务、对台事务、档案、地方志、扶贫开发、防震减灾、机关事务管理等工作继续加强。社会治理水平不断提高。推进社区“三化”建设，城市社区全部实现网格化管理。建立完善多元化纠纷解决机制，成立市行政调解委员会和仲裁委员会。“六五”普法、法律援助、人民调解、社区矫正和安置帮教工作进一步强化。郎溪、绩溪被评为全省司法所建设示范县。加强立体化治安防控体系建设。流动人口居住证制度开始实施。积极创新群众工作体制机制，深入开展领导干部开门接访、带案下访等活动，一批信访积案得到化解。大力开展安全生产检查和“打非治违”行动，关闭非煤矿山25座，各类安全生产事故起数下降35.2%。6个县市成为省食品药品安全示范县。地质灾害防治进一步加强。建成全国综合减灾示范社区3个。国防教育、国防动员、人民防空工作不断提升，连续三届荣获省双拥模范城称号，驻宣部队和民兵预备役人员在抗灾救灾、服务地方发展中作出了重要贡献。

（五）扎实推进自身建设，推动科学发展的能力进一步增强。坚持依法行政。修订出台市政府工作规则，坚持市政府全体会、常务会、市长工作例会和规委会、土委会、重点项目评委会等制度，促进民主科学决策。自觉接受市人大、市政协和各民主党派的监督，办理人代会议案和人大代表建议64件、政协委员提案和建议182件。积极推进政务公开，开通宣城政务微博，新闻发布和网络政民互动常态化。优化政务服务。实施新一轮行政审批制度改革，市级行政审批事项精简73.1%，办理时限缩短65.2%。民主考评和政风行风评议活动继续深化，行政效能和政务服务水平得到提升。全面落实国家和省促进经济发展的各项政策，配套出台促进实体经济发展、加快民营经济发展等政策措施，组织开展“三进三解三推动”和“进百家企业、解千个难题”等活动。完善推进机制。加强市领导联系重大项目和重点工作、分层分级调度、项目督查考核、联点共建等制度，实行制表上墙、挂图作战。健全征迁拆违工作机制，全面推开征迁拆违工作。强化应急处置。及时出台保龙头、保企业、保重点的政策措施，有效缓解了禽流感疫情冲击，促进了家禽业稳定发展。快速应对、有效抗击两次洪涝灾害，扎实推进灾民生活安置、生产自救和灾后重建工作。全面发动、科学应对历史罕见的持续高温旱灾，有力保障了群众生活生产。加强廉政建设。严格执行中央“八项规定”和省委、市委“三十条规定”，加强财政支出管理，“三公经费”下降18.2%。强化政府投资建设项目监管，对497个政府投资项目实行预算审核和决算审计，节约资金3亿元。市招投标中心荣获“全国建筑市场与招投标行业先进单位”称号。

各位代表，过去一年的成绩来之不易。这是省委、省政府和市委坚强领导的结果，是市人大、市政协监督支持的结果，更是全市人民同心同德、顽强拼搏的结果。在此，我代表市人民政府，向全体人大代表、政协委员，向全市广大工人、农民、知识分子、干部、离退休老同志，向驻宣部队、武警和消防官兵、政法干警，向各民主党派、工商联、无党派人士、各人民团体，向所有关心、支持、参与宣城建设发展的同志们、朋友们，表示崇高的敬意和衷心的感谢！

我们也清醒认识到，经济社会发展还存在不少困难和问题。经济运行下行压力较大，一些企业生产经营困难，投资意愿下降，部分指标和项目未能实现预期目标；产业层次不高、创新能力不强、服务业落后、“大、优、高”项目缺乏等问题在转方式、调结构中进一步凸显；市本级总量不大、辐射力不强的状况依然存在；资源环境、生产要素约束偏紧，污染治理难度加大；财政收支矛盾和债务压力加大，医疗、教育等民生问题有待更好地解决；影响和谐稳定发展的因素仍然不少；政府职能越位缺位、“四风”现象不同程度地存在。对此，我们一定高度重视，找准症结所在，采取有效措施，努力早日化解。

二、2014年工作目标和重点任务

2014年是全面贯彻落实党的十八届三中全会精神、全面深化改革的开局之年，也是完成“十二五”规划目标任务的关键一年。今年，宏观政策将趋于稳定，经济升级、转型发展的要求更为迫切，经济增长将在适度区间运行。我市加入南京都市圈后，对接长三角更为直接紧密，皖江城市带等战略带动作用进一步显现。面对国际国内经济调整升级、增长换档调速的新态势，我们要认清仍可作为的宏观大势，牢牢把握发展的主动权，增强忧患意识，树立底线思维，坚持奋发有为，把改革贯穿于经济社会发展全过程，振奋精神、抢抓机遇，促进经济持续健康发展、社会和谐稳定。今年政府工作的总体要求是：全面贯彻落实党的十八大、十八届三中全会和中央、全省经济工作会议精神，坚持稳中求进总基调，弘扬改革创新主旋律，把握“好为标准，快字当先”工作要求和"11331"发展思路，大力推进“六年并进、六城同创”活动，确保发展速度高于全省平均水平，质量效益稳步提升，为圆满完成“十二五”规划目标、全面建成小康社会奠定更加坚实的基础。

建议全市经济社会发展的主要预期目标是：地区生产总值增长10.5%；规模以上工业增加值增长14%；服务业增加值增长10%；财政收入增长12%；固定资产投资增长18%；社会消费品零售总额增长13%；进出口总额增长10%；居民消费价格涨幅控制在3.5%左右；城镇新增就业3.3万人；城镇居民人均可支配收入增长与经济增长基本同步，农民人均纯收入增长11%以上；确保完成省定节能减排任务。

重点做好六个方面工作：

（一）以“项目提质提效年”和“招商合作年”为抓手，千方百计加快项目建设

大力推进项目提质提效。深入研究国家产业政策、投资导向和市场动向，围绕主导产业、战略性新兴产业、现代服务业、新型城镇化、生态建设等领域，深入谋划和引进实施一批“大、优、高”项目，力求更多项目进入国家和省“盘子”。落实投资强度、容积率和建筑密度、预期亩均税收等要求，促进土地资源节约集约利用，提高项目投资效益。坚持重点项目分级分类调度和市领导联系重大项目等制度，加大项目推进力度，确保列入省"861"项目数增长10%以上，投资计划完成比率居全省前列；实施亿元以上项目500个，完成投资500亿元以上。

不断提高招商合作水平。创新招商理念，优化招商方式，紧盯国内外500强、全球跨国集团和行业龙头企业，加强与港澳台等地区经贸交流，深化与央企和知名民企合作。优化、完善专业招商机构和招商工作机制，加快推进市场化招商、小分队招商、以商招商、精准招商与全民招商的有效结合。强化信息摸排、跟踪、反馈和处置，制定“一对一”招商方案，开展“点对点”招商，确保重要信息不放过、重大机遇不错过。力争引进重大项目50个以上，其中到位5000万美元以上外资项目5个，亿元以上项目到位省外资金增长18%、实际利用外商直接投资增长15%。科学构建招商引资考核评价体系，坚持项目准入标准，完善项目评审制度，引导项目合理布局，加快符合要求项目的落地进度，提高资金到位率和产业集中度。

努力破解要素瓶颈制约。强化用地保障。坚持争取增量和盘活存量并重，力争报批建设用地规模不低于上年水平，进一步盘活批而未用和低效、闲置用地。加强标准化厂房建设和管理，着力提高使用效率。继续开展国土资源执法和节约集约模范市（县）创建活动。全面推行地票交易制度。强力推进征迁工作，努力让土地等项目。强化融资保障。有效利用金融工具，大力推进政银企合作，做大做强融资性担保机构。加大银行信贷对实体经济的支持，优化投放结构，力争新增贷款135亿元。加强企业上市、中小企业债券和股权、商标专用权质押等融资工作，发行市国投公司二期债券，力争中期票据、短期融资券、私募债发行有较大进展，3-5家企业在“新三板”和省股权交易中心挂牌，完成直接融资30亿元。

（二）以新型工业化和文化旅游产业为核心，加速构建现代产业体系

扎实开展“工业突破年”活动。以打造百亿企业、千亿产业为主攻方向，以开发区为龙头，加大工业投入，加强产业集聚。做大做强支柱产业。突出装备制造、新材料和食品医药产业，制定中长期发展规划，实行差异化扶持政策。择优确定一批终端产品，强化产业增链、补链。培育壮大企业群体。实施重点企业培育"136151"计划，力争培育产值超100亿元企业1户、超50亿元3户、超20亿元6户，力争100家小微企业进入规模以上企业，着力培育50户专精特新企业和100户成长型企业。推进工业化与信息化深度融合，重点建设100户“智慧企业”、8户“两化融合”示范企业和2个“智慧园区”。加强开发区建设。进一步创新创优开发区体制机制，激发发展动力。坚持发挥开发区产业集聚、招商承接功能，促进要素、项目集中。加快推进宣城开发区世行贷款基础设施、中鼎发动机等重点项目建设，力争新签项目100个、开工50个、竣工投产30个。研究增强开发区综合优势的措施办法，加快县市区开发区主导产业发展。宣城、广德开发区争创国家级开发区。扩大工业有效投入。确保工业投资增长18%。实施重点项目“三百”计划，新开工、投产、达产项目各100个。市工业投资公司进入实体运作。强化企业帮扶。继续实行市领导联系帮扶措施，加快中小企业服务中心建设，开通96871企业服务热线，建立“企业服务日制度”，帮助企业排忧解难。发挥宣城职教联盟作用，深化产教融合、校企合作，推广“订单”模式，提高培训实效。开展与皖北等劳务输出地的对接，拓展劳动力来源。

突出发展文化旅游产业。扎实开展“文化旅游发展提升年”活动，力争文化产业增加值、旅游总收入增长18%。设立文化旅游发展引导资金，实施文化旅游产业“八大工程”三年行动计划，建设一批文房四宝城市品牌提升、古建筑古遗址保护利用、休闲养生乡村旅游的重点项目，着力打造“六个集聚区”。继续推进市本级旅游项目"6521"工程，启动敬亭山文化旅游度假区建设，开展金梅岭、太极洞、大桃花潭、宣酒文化博览园等景区5A创建工作。加快宣州昆山湖生态旅游度假区、郎溪中华茶博园、广德卢湖竹海休闲度假区、宁国青龙湾、绩溪百里历史文化生态走廊、旌德东黄山国际灵芝养生度假区等项目建设。创新营销方式，开展全域一体化营销，打造“山水诗乡、多彩宣城”城市形象品牌，创建国际性旅游目的地城市。推进文教创意产业园基础设施建设，加快现有项目进度，招商新入驻一批项目。

加快发展现代服务业。大力发展商贸服务业。修订市区商业网点布局规划，重点打造中央商务区、城市特色街区和城市综合体。规划建设物流公路港。推进双桥物流园区健康发展，完善道路路网等基础设施建设，开工建设羽绒产业园、农机市场等项目，加强错位引导，合理引入新项目。全面启动中小商贸流通企业服务体系建设试点。推进菜市场、农贸市场的新建和改造。加强“新网工程”建设，改造提升日用消费品、农资等六大经营服务网络体系。促进金融业加快发展。规划建设金融集聚区。引进2家以上金融机构，力争宣州、广德村镇银行开业，推进旌德村镇银行组建。促进农商行加快发展。提升小额贷款公司、融资性担保公司发展水平。加强金融生态县创建。培育新型服务业态。加快发展电子商务，壮大淘宝网宣城馆、宁国馆等主体，努力打造电子商务、实体商贸、物流配送相融合的电商产业集聚园区。举行首届工业品暨消费品展示展销会。大力发展会展经济、文化创意、服务外包、健康养老、信息服务等产业。

壮大提升现代农业。构建现代农业经营体系。发展农村合作经济，出台促进新型农业经营主体发展的意见。争创1-2家国家级龙头企业。发展农民专业合作社400家以上，争创省级示范社7家。创建省级示范家庭农场50家、市级100家。完善土地承包经营权流转机制，加快土地承包经营权向新型农业经营主体流转，新增流转面积18万亩。探索发展土地股份合作社。推进农业产业化发展。加快农业科技园、农业产业化示范区和现代农业示范区建设。重点抓好30个农产品加工项目建设，力争农产品加工产值增长15%。积极开展“三品”认证，争创省级以上名牌（农）产品5个以上。健全市、县农产品质量安全监管体系，确保市检验检测中心建成运行。大力开拓农产品市场，推进农超、农批对接和农业物联网建设。发展农业特色产业。稳定粮食生产。重点培育畜禽、蔬菜、水产、林特产品等优势产业基地。实施茶产业提升计划，新改建茶园2万亩，建成标准化茶园1万亩以上，重点培育2-3户省级茶叶龙头企业。新增烟叶种植面积3万亩，力争收购总量突破50万担。大力发展林下经济，建成示范点10个。推进道地中药材产业化基地建设。新建1000亩市区保障性蔬菜基地。

（三）以建设皖苏浙交汇区域中心城市为目标，加快推进新型城镇化和生态文明建设

有序推进农业转移人口市民化。加快户籍制度改革，研究制定全面放开县城以下户籍和有序放开市区户籍的办法，全面推行居住证制度，切实解决已转移到城镇就业的农业转移人口落户问题，扩大建成区人口规模。逐步把进城落户农民纳入城镇住房和社会保障体系，稳步推进城镇基本公共服务常住人口全覆盖。

加速构建科学合理的城镇布局。健全城乡规划体系，加强城市总体规划修编和城市规划项目编研工作，强化规划的引导和控制作用。全面实施新型城镇化"11134"工程，促进中心城市和城镇合理分工、功能互补、协同发展。推进市区一体化建设，加快市本级发展。优化城镇空间结构，扎实开展宣城、广德开发区产城一体试点。推进行政区划调整和区域优化，积极培育县级中等城市。强化产业支撑，提高县域承载能力，壮大县域经济。加快省扩权强镇试点镇和中心镇发展。坚持错位发展、传承文化、突出特色，加快培育一批美丽城、镇、村。

提升中心城区建设管理水平。着力“补功能、提品位、优环境”，大力推进“城市建设年”活动，打造山水相依、人文相伴、生态相连、宜居宜业宜游的城市特色。推进中心城区178个项目建设，确保完成投资150亿元。完善城市路网，续建和新改建城市道路79条。加快合工大宣城校区二期、市国防训练基地、北门棚户区改造等重点项目建设进度。继续实施老旧小区和背街后巷综合整治、河道清淤截污等工程。启动宛溪河综合整治二期及周边棚户区改造项目。加快宝城路改造、水阳江大道闭合段工程建设。完善市区消防站布局，规划建设宣城开发区、宣州开发区和城东消防站。加快推进彩金湖生态新区建设，争取建成关庙水库，开工建设文景路、创业路、圣俞路、安置区和市人民医院新院区项目。加快地理空间框架建设。完成“数字城管”二期项目。进一步理顺市区城市管理体制，推进城管执法重心下移。实施市本级存量违法建设三年拆除行动。加大垃圾消纳和“牛皮癣”、渣土、噪音等城市痼疾治理力度，提升管理精细化水平。巩固文明创建成果，健全文明创建工作长效机制。

继续开展“交通建设会战年”活动。确保完成投资80亿元，加快构建“市县一小时”、“县乡半小时”交通圈。确保宁绩高速年内完工，加快宁千、溧广、宣南铜高速建设，开工建设宣狸、广宁高速和广德至祠山岗一级公路项目，加快推进宣泾线等10个国省干线一级公路续建工程。实施100公里以上国省道路面改善。完成芜申运河定埠段航道疏浚和港口工程，启动水阳江航道和宣州综合码头建设。加快宣城、郎溪、宁国等客运枢纽站建设。配合完成合福铁路宣城段建设扫尾和商合杭铁路前期工作。做好皖赣铁路扩能改造工程征迁和服务工作。开工建设杭黄铁路和宣城火车站站场。力争开工建设芜宣机场。

加快建设美好乡村。完成县市区村庄布点规划，加强中心村规划建设，完成57个省级中心村建设任务，全面建设美好乡村示范带。着力改善农村人居环境，以“三线四边”为突破口，大力开展“四治理一提升”行动。继续实施农村清洁工程和危房改造工程，健全垃圾处理全覆盖长效机制，实现减量化、市场化、常态化。

确保国家生态市等创建工作取得实质性进展。力争国家环保模范城市创建通过技术评估，郎溪、广德、泾县和旌德创成国家生态县，支持宁国、绩溪开展全国生态文明建设试点工作。推进国家森林城市创建工作，创成省级森林城市2个、城镇18个、示范村庄30个。大力实施森林增长工程，新造林13.65万亩，建设森林长廊示范段60公里。全力推进国家园林城市创建工作，年内基本达到创建标准。加快市区植物园、九曲公园、明镜湖公园、麒麟山公园及清溪河景观带建设。推进绿化生态城区和绿色建筑建设。加强节能减排工作。强力推进污水处理设施配套管网和省级以上工业园区、重点乡镇污水处理厂建设。积极防治大气污染，加强工业废气、机动车尾气、城市扬尘等治理。加快规模化畜禽养殖污染防治设施建设。大力防治农业面源污染，保持生物多样性。继续开展环保绿剑行动，确保区域环境安全。加强南漪湖周边环境保护和控制。实行最严格水资源管理制度，鼓励企业开展节水和清洁生产。加强饮用水水源地的保护。

加强水利、电力等基础设施建设。全面完成水阳江开卡、三板闸、双桥联圩城市规划区堤防除险加固等工程。开工建设城东联圩宛溪河治理二期、敬亭圩除险加固工程。力争开工青弋江治理、双塘排涝站项目。启动青弋江引水入城、水阳江拦河筑坝、城市内河整治三大工程。推进青弋江灌区、港口湾水库灌区和牛岭、郎源、汤村、扬溪源、粮长门、凤凰山、万家、浣溪等水库建设前期工作。继续实施小型水库除险加固和中小河流治理，加快农村饮水安全工程建设，推进以“八小”工程为重点的小型水利工程改造提升。加强河道采砂整治与管理。积极探索小型水利工程管护体制机制创新。续建、新建18个110千伏以上主电网项目，继续加强配电网和农网改造，力争完成投资比上年翻一番。加强“智慧城市”建设，加快推动光纤入户工程，开通4G网络。启动市级政务云计算中心和党政数据交换平台建设。

（四）以全面深化改革为动力，推进创新驱动发展

力争重要领域和关键环节改革取得成效。深化行政体制改革。按照精简、统一、效能的原则，稳步推进政府机构改革。完成市县工商、质监行政管理体制调整和食品药品监管体制改革。继续支持广德“省直管县”体制改革试点工作。改革创新社会组织建设，实施政府向社会组织购买服务。推进事业单位分类改革，加快构建公益服务新格局。深化财税体制改革。继续做好“营改增”扩围试点。编制国有资本经营预算，推进预算绩效管理。加强政府债务管理，有效防范债务风险。深化农村综合改革。积极推进统筹城乡土地使用制度综合改革试点进程，完善城乡土地交易市场运作制度，探索开展农村宅基地制度改革等工作。完善集体林权制度配套改革。支持宣州、广德省级农村综合改革示范试点，推进土地承包经营权确权登记颁证工作。推进郎溪城乡一体化综合配套改革。深化社会领域改革。深化文化体制改革，加快国有经营性文化单位公司制、股份制改造。继续深化医药卫生体制改革，扩大新农合大病保险试点。巩固和完善基层、县级公立医院综合改革，推进城市公立医院改革。有序疏导民生和资源价格矛盾。

增强自主创新能力。加快创新型城市建设。积极打造创新平台，新组建省级工程技术研究中心3家、企业技术中心4家，组建皖江禽产业研究院。加快宣城开发区科技园等孵化器建设，加快引进转化一批科技成果和项目。加强与合工大等高校院所合作，确保产学研合作取得新成果。加强创新主体建设，实施省级以上科技计划项目70个，力争新增高新技术企业33家、省创新型试点企业3家。提升知识产权创造和应用能力，发明专利申请量增长30%以上。争创中国驰名商标1件、省著名商标20件、安徽名牌产品10个、省卓越绩效奖5个以上。加强国家级、省级农业和服务业标准化示范（试点）项目建设。完善和落实人才政策，着力引进和培养高精尖专业人才及高级技师。

深入开展全民创业。按照“非禁即入”的原则，推行负面清单管理，支持民间资本进入基础设施、社会事业和公共服务等领域。全面落实国家、省支持政策，进一步放宽经营条件、降低门槛，促进全民创业，力争1000户“个转企”。积极争创省创业型城市。积极发展混合所有制经济，促进民间资本和国有资本、集体资本融合。大力倡导尊重创业、尊重劳动、尊重企业家的风尚，营造重商、亲商、安商和支持创业者、褒扬成功者、宽容失误者的社会氛围。

大力发展开放型经济。加强对外区域合作，积极参与南京都市圈建设与发展。积极对接、利用上海自贸区，主动承接产业和服务辐射。打造“宣城出口基地”品牌，实施外贸“双百”工程，重点扶持100户年进出口额超300万美元企业，培育100户新发生进出口实绩企业。加大国外技术、管理、资金、人才、团队等引进力度，强化与国际市场对接。力争宣城海关、出入境检验检疫局“开关开检”。大力支持企业“走出去”开拓国际市场、开发国外资源。

（五）以提升群众幸福指数为出发点和落脚点，尽心竭力保障改善民生

提升社会保障水平。完善收入分配机制，努力提高居民收入。坚持守住底线、突出重点、完善制度、引导舆论，扎实推进民生工程建设。完善创业扶持政策，促进高校毕业生、农民工和困难群体就业。继续完善社保制度，强化基金扩面征缴，提高城镇职工、居民基本医疗保险政策范围内支付比例。加快社会保障服务中心建设。完善养老服务和农村留守儿童、妇女、老人关爱服务体系。继续做好被征地农民等养老保障工作。强化城乡低保、农村敬老院规范化管理。健全临时救助制度，启动市救助管理站建设。健全住房保障和供应体系，推进廉租房、公租房并轨运行。加强保障性安居工程建设，开工建设保障性住房16584套，市本级开工安置房4100套、竣工交付6000套。继续扩大住房公积金制度覆盖面，完善提取、使用、监管机制。认真办好面向社会征集的10件民生实事。

加快发展社会事业。确保市图书馆、规划馆、体育馆建成。继续推进村级文化广场、农民文化乐园和重点示范村文化活动中心建设。完成20个乡镇、村、社区公共电子阅览室建设。申创中国书法城。开展第一次全国可移动文物普查，加强“非遗”申报工作。加强皖南花鼓戏等地方传统文化的保护与传承。启动市博物馆新馆、中国文房四宝博物馆、美术馆“三馆合一”建设工程。开展“百场文艺下基层”和节假日群众文化活动。启动第二期学前教育三年行动计划。推进基础教育信息化建设，加快市区中小学建设与发展，取消重点班、限制大班额，促进义务教育资源均衡配置。完成235所义务教育学校标准化建设任务。推进宣城中学打造皖南名校工程。加快发展现代职业教育。确保市示范性综合实践基地竣工。深入开展全民健身运动和竞技体育，力争在省第十三届运动会上取得好成绩。进一步加强卫生基础设施和农村三级卫生服务体系建设，健全基层医疗机构和村卫生室运行机制。强化重大疾病防控和卫生应急处置。建成市新生儿疾病筛查中心。继续深化人口计生“村为主”工作机制，加强城市和流动人口计生工作网格化管理，完善利益导向机制，提升优质服务水平，确保主要指标位居全省前列。继续开展民族团结进步创建活动，依法管理宗教事务。加强残疾人组织规范化和市残疾人康复中心建设。加快马山生态陵园和烈士陵园建设。加强农村公益性公墓建设管理。推进新一代多普勒天气雷达项目。扎实开展第三次全国经济普查工作。充分发挥工会、共青团、妇联等人民团体作用。做好外事侨务、对台、物价、防震减灾、档案、地方志、烟草等工作，推动妇女、儿童、老龄和红十字、慈善福利事业加快发展。

深入推进社会治理。继续加强综合治理“十大体系”建设。深化城市社区管理体制改革试点，推进社区信息化、标准化建设和网格化管理。健全群众工作网络，推动“信、访、网、电”一体化。落实矛盾纠纷排查调处、信访代理、社情分析研判等机制，完善司法救助、法律援助制度，开展争创全省社区矫正示范县活动，进一步强化安置帮教工作，实行人民调解、司法调解、行政调解与信访工作协调联动，及时有效防范和化解争议纠纷。扩大“六五”普法覆盖面，积极争创省级法治文化建设示范城市（县）和示范基地。深入开展领导干部接访、下访等活动，畅通和规范群众诉求表达渠道，推进信访积案化解，维护群众合法权益。完善流动人口和特殊人群管理服务。重视媒体、网络在了解社情民意、反映群众诉求、教育引导群众中的作用，增强新媒体时代社会沟通能力。

强化公共安全体系建设。严格落实安全生产责任，继续开展重点行业领域安全生产隐患排查整治，加大“打非治违”力度，有效遏制重特大安全事故。强化产品质量安全监管，全面推行首席质量官制度。强化食品药品安全监管，争创国家级食品药品安全示范县。加强校车安全管理。健全应急预案体系，加强应急指挥平台和应急队伍建设，切实提高防灾减灾救灾和应对突发事件能力。完善立体化、数字化社会治安防控体系和公共安全体系，防范和依法惩治违法犯罪活动，深化平安宣城建设。加强国防教育、国防动员、民兵预备役、人民防空和双拥优抚安置工作，巩固发展军政军民团结。积极争创全国双拥模范城。

（六）以提高公信力和执行力为重点，努力建设法治政府和服务型政府

切实转变政府职能。尊重市场规律、尊重群众创造，创新政府管理，强化政府公共服务、市场监管、社会管理、环境保护等职责，做到少审批、少收费，实现高度透明、高效服务。接好中央、省下放的审批事项，市级行政审批事项再精简10%。推行网上审批和“一表制”、“一费清”，完善并联审批、“绿色通道”等制度，加强行政审批绩效管理。创新政务公开方式，深化政务公开工作。加强网络政民互动、市长热线和政务微博发布工作。完善新闻发布和网络新闻发言人制度。大力推进政务服务标准化体系建设，强化招商引资帮办服务，完善乡村为民服务全程代理。严格落实分管责任制、牵头负责制、限时办结制和责任追究制，加大效能建设明察暗访和电子监察力度，不断提升服务效能。

切实提高依法行政水平。健全行政决策程序，完善听取意见、听证、风险评估、合法性审查、集体决定等制度。强化决策执行监督，完善决策实施后评估和纠错制度。认真执行市人大及其常委会决议、决定，自觉接受人大依法监督。积极支持政协履行政治协商、民主监督、参政议政职能。提高人代会议案、人大代表建议和政协委员提案办理质量。实行市政府负责同志领衔办理议案和重要提案制度。充分听取各民主党派、工商联和社会各界人士的意见建议。深入推进依法行政示范单位创建活动，健全行政复议案件审理机制。整合执法主体，推进综合执法，规范执法自由裁量权，强化执法监督，进一步落实行政执法责任制，做到严格规范公正文明执法。

切实践行群众路线。扎实开展党的群众路线教育实践活动，坚决反对形式主义、官僚主义、享乐主义和奢靡之风。认真落实关于改进工作作风、密切联系群众的各项规定，大兴调查研究之风，切实改进会风文风，着力解决群众反映强烈的突出问题。深入开展政风行风评议活动，加强政务督查督办、行政监察和投诉受理工作，强化行政问责，督促职能部门依法履职尽责，做到令行禁止，严肃查处违反规定、损害发展环境的行为。始终坚持务实勤政，做到知实情、讲实话、干实事、求实效。

切实加强廉政建设。加强理想信念和从政道德教育，深入推进惩治和预防腐败体系建设，以干部清正、政府清廉、政治清明的实际成效取信于民。加强对重点工作、政策落实情况的监督检查，强化政府投资项目标后监管和工程量变更管理，继续加大对政府性资金使用情况和领导干部经济责任审计力度。坚持有案必查、有腐必惩，严肃查处各类违法违纪案件和顶风违纪的典型，着力解决发生在群众身边的腐败问题，坚决纠正损害群众利益的不正之风。牢固树立长期过紧日子的思想，厉行节约，反对铺张浪费，严格控制一般性支出，进一步压缩“三公”经费。推进市直30家单位预决算及“三公”经费信息公开，把有限的公共财力用在最需要的地方。巩固公务用车专项治理成果，实施公务用车改革。

各位代表！百舸争流千帆竞发，奋勇当先时不我待。加快宣城发展，重任在肩；做好政府工作，责任巨大。让我们在中共宣城市委的坚强领导下，凝聚全市人民的智慧和力量，弘扬“千锤百炼、精雕细作、刚柔相济、百折无损”的当代宣城干部精神，坚定信心、进取作为，开拓创新、务实笃行，奋力谱写“中国梦”的宣城篇章！
